# Efficacy of *Lactococcus lactis* subsp. *lactis* LY-66 and *Lactobacillus plantarum* PL-02 in Enhancing Explosive Strength and Endurance: A Randomized, Double-Blinded Clinical Trial

**DOI:** 10.3390/nu16121921

**Published:** 2024-06-18

**Authors:** Mon-Chien Lee, Yi-Ju Hsu, Mu-Tsung Chen, Yi-Wei Kuo, Jia-Hung Lin, Yu-Chieh Hsu, Yen-Yu Huang, Ching-Min Li, Shin-Yu Tsai, Ko-Chiang Hsia, Hsieh-Hsun Ho, Chi-Chang Huang

**Affiliations:** 1 Graduate Institute of Sports Science, National Taiwan Sport University, Taoyuan 333325, Taiwan; kurt0710@ntsu.edu.tw (M.-C.L.); ruby780202@ntsu.edu.tw (Y.-J.H.); 2 Center for General Education, Taipei Medical University, Taipei 110301, Taiwan; 3 Committee on General Studies, Shih Chien University, Taipei City 104, Taiwan; bruce@g2.usc.edu.tw; 4 Functional R&D Department, Research and Design Center, Glac Biotech Co., Ltd., Tainan City 744, Taiwan; vic.kuo@glac.com.tw (Y.-W.K.); jiahung.lin@glac.com.tw (J.-H.L.); jim.huang@glac.com.tw (Y.-Y.H.); chingmin.li@glac.com.tw (C.-M.L.); sam.ho@glac.com.tw (H.-H.H.); 5 Research Product Department, Research and Design Center, Glac Biotech Co., Ltd., Tainan City 744, Taiwan; yuchieh.hsu@glac.com.tw (Y.-C.H.); shin-yu.tsai@glac.com.tw (S.-Y.T.); shawn.hsia@glac.com.tw (K.-C.H.); 6 Tajen University, Pingtung 907101, Taiwan

**Keywords:** probiotics, *Lactobacillus plantarum* PL-02, *Lactococcus lactis* subsp. *lactis* LY-66, explosive strength, muscle health, exercise

## Abstract

Probiotics are posited to enhance exercise performance by influencing muscle protein synthesis, augmenting glycogen storage, and reducing inflammation. This double-blind study randomized 88 participants to receive a six-week intervention with either a placebo, *Lactococcus lactis* subsp. *lactis* LY-66, *Lactobacillus plantarum* PL-02, or a combination of both strains, combined with a structured exercise training program. We assessed changes in maximal oxygen consumption (VO_2max_), exercise performance, and gut microbiota composition before and after the intervention. Further analyses were conducted to evaluate the impact of probiotics on exercise-induced muscle damage (EIMD), muscle integrity, and inflammatory markers in the blood, 24 and 48 h post-intervention. The results demonstrated that all probiotic groups exhibited significant enhancements in exercise performance and attenuation of muscle strength decline post-exercise exhaustion (*p* < 0.05). Notably, PL-02 intake significantly increased muscle mass, whereas LY-66 and the combination therapy significantly reduced body fat percentage (*p* < 0.05). Analysis of intestinal microbiota revealed an increase in beneficial bacteria, especially a significant rise in *Akkermansia muciniphila* following supplementation with PL-02 and LY-66 (*p* < 0.05). Overall, the combination of exercise training and supplementation with PL-02, LY-66, and their combination improved muscle strength, explosiveness, and endurance performance, and had beneficial effects on body composition and gastrointestinal health, as evidenced by data obtained from non-athlete participants.

## 1. Introduction

Regular exercise has been extensively studied and scientifically validated for its numerous health benefits across a range of physical conditions, including cardiovascular disease, lung disease, metabolic syndrome, cancer, and sexual dysfunction [1]. Among the array of benefits conferred by exercise, it is particularly noteworthy that it can enhance maximal oxygen uptake, thereby fortifying cardiovascular robustness. Maximal oxygen uptake (VO_2max_), the maximum rate of oxygen consumption during intense and maximal exercise, serves as a crucial measure of cardiovascular health and aerobic endurance [2,3]. The ability to transform nutrients into adenosine triphosphate (ATP) via aerobic pathways is directly linked to how effectively oxygen is utilized by the muscles, thereby enhancing athletic performance and supporting cardiovascular health, potentially reducing the risk of developing atherosclerosis [4]. Exercise plays a pivotal role in positively influencing the musculoskeletal system by enhancing muscular strength, endurance, and flexibility. These benefits not only help mitigate muscle loss but also serve as preventive measures against falls in the elderly [5]. Furthermore, regular physical activity significantly contributes to enhanced social well-being, improved quality of life [6], and increased longevity [7]. It is noteworthy that exercise can also be associated with elevated self-esteem and a reduced risk of anxiety in younger individuals [8,9]. Despite these advantages, many individuals experience post-exercise fatigue, which can hinder the maintenance of a regular exercise routine. This form of fatigue typically arises from exercise-induced muscle damage (EIMD). During the initial phases of muscle damage, mechanical trauma triggers an acute inflammatory cascade, resulting in the release of pro-inflammatory cytokines such as interleukin 6 (IL-6), tumor necrosis factor-alpha (TNF-α), and IL-1β, alongside heightened levels of creatine kinase (CK) and myoglobin [10,11]. Beyond the acute inflammatory response, prolonged endurance exercise precipitates oxidative and metabolic stress, further exacerbating inflammation [12,13]. These physiological alterations result in a temporary decrease in muscle strength and performance. To modulate the dysregulated inflammatory response while continually optimizing athletic performance and preserving health, athletes have increasingly turned to dietary supplements in recent years. Among these, the utilization of probiotics for health enhancement and performance augmentation has attracted growing interest.

Scientists have previously proposed the concept of the “gut–muscle axis”, suggesting a connection between the gut microbiota and muscle cells. It is believed that the gut microbiota, composed of four main phyla including Firmicutes, Bacteroidetes, *Proteobacteria*, and *Actinobacteria*, may play a role in producing beneficial nutrients such as short-chain fatty acids (SCFAs) that can impact muscle cells [14,15]. The human gut microbiota consists of an incredibly vast number of microorganisms, with over 10^14^ cells, which is approximately ten times the number of bacterial cells compared to the total count of human cells. Furthermore, the gut microbiota has a genome size that is 150 times larger than the human genome [16]. This highlights the significant presence and potential impact of the gut microbiota on the human body’s normal homeostasis. However, the specific mechanisms by which the gut microbiota regulates the metabolism of muscle cells are still not fully understood. Further research is needed to uncover the intricate workings of the gut–muscle axis and the specific interactions between the gut microbiota and muscle cells.

A human study reported that the probiotic strain *Bacillus coagulans* demonstrated potential in reducing exercise-induced muscle damage [17]. Another study focused on a probiotic strain isolated from humans, *Bifidobacterium longum* subsp. *longum* OLP-01, which was found to enhance endurance running distance in middle- and long-distance runners [18]. Furthermore, this probiotic strain was associated with increased muscle grip strength and a reduction in fatigue induced by exhaustive exercise [19]. Additionally, our previous animal study investigated two probiotic strains, *Lactococcus lactis* subsp. *lactis* LY-66 and *Lactobacillus plantarum* PL-02, which were isolated from an Olympic elite athlete. These strains were found to significantly elevate grip strength and endurance in tested mice. The mechanisms underlying these effects were proposed to involve improved fatty acid metabolism and increased glycogen storage levels in the liver and muscles, leading to enhanced exercise performance in the mouse model [20]. However, whether these two probiotic strains can similarly promote exercise performance in humans remains uncertain. While these studies show promising findings regarding the potential benefits of specific probiotic strains on exercise-related outcomes, it is important to note that further research is needed to validate and understand the precise mechanisms involved. Additionally, individual variations, the specific strains and dosages of probiotics, and other factors should be considered.

The aim of this study was to investigate the effects of PL-02, LY-66, and their combined supplementation on exercise performance, resilience to post-exercise muscle strength loss, and gastrointestinal health. Initially, we measured participants’ body composition, blood biochemical markers, exercise performance, and maximal oxygen consumption (VO_2max_) both prior to and following the probiotic intervention. For the assessment of muscle strength loss following exercise-induced muscle damage (EIMD), we conducted countermovement jump (CMJ) and isometric mid-thigh pull (IMTP) tests. Additionally, to measure the extent of muscle damage and inflammation, we analyzed specific biomarkers in the blood. To examine changes in the gut microbiota, we employed next-generation sequencing (NGS) technology pre- and post-intervention, providing a comprehensive analysis of the microbiota’s response to probiotic supplementation.

## 2. Materials and Methods

### 2.1. Experimental Test Samples

The probiotic strains of *Lactococcus lactis* subsp. *lactis* (LY-66) (BCRC 911055 = CGMCC 21838) and *Lactobacillus plantarum* (PL-02) (BCRC 911012 = CGMCC 20485) were isolated from the gut of a weightlifting Olympic gold medalist and obtained from Glac Biotech Co., Ltd (Tainan, Taiwan). [20]. The genetic sequences of these probiotic strains have been determined through sequencing techniques. Subsequently, the strains were encapsulated to produce a uniformly standardized dry beverage in appearance.

### 2.2. Participants

The Harvard calculator (http://hedwig.mgh.harvard.edu/sample_size/size.html, accessed on 12 February 2022) was used to determine the sample size, assuming a parallel design with a significance level of 0.05, a power of 0.9, and a minimal detectable difference (following our pilot study) of 0.36 for the difference. A total of 84 patients entered this study. To account for the possibility of subject dropout, we added one person to each group to ensure minimum sample size requirements. A total of 88 healthy non-athlete adults aged 20–40 were included in this study, among which 44 participants were males and 44 were females. The exclusion criteria were as follows: smokers, cardiovascular disease, high blood pressure, BMI > 27, metabolic disease, asthma or within 6 months, those who are unable to engage in sports due to physical or neuromuscular injuries, those who have taken anti-inflammatory and analgesic drugs or probiotic-related products in the past month, students or related stakeholders of the program investigator. The Institutional Review Board of Landseed International Hospital (Taoyuan, Taiwan; LSHIRB No. 21-042-A2) reviewed and approved the conduct of this clinical study. The trial was as registered at clinicaltrials.gov as NCT06092723 on 23 October 2023. Furthermore, this study was executed by following the principles of the Declaration of Helsinki.

### 2.3. Experimental Design and Content

**Figure 1:**
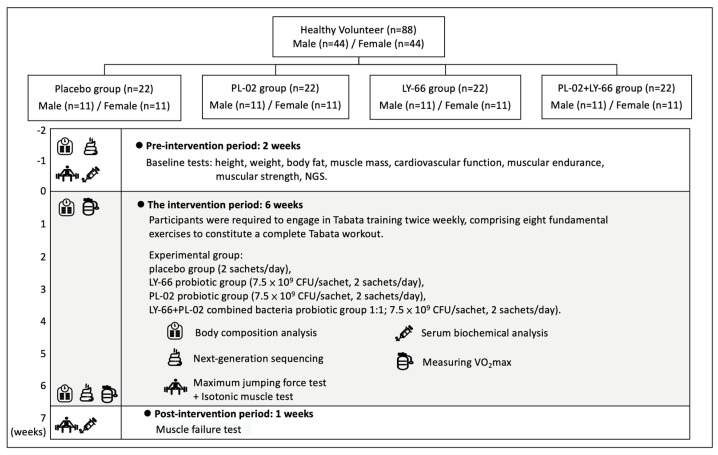
Experimental procedure description.

A total of 88 healthy recruited participants were randomly and double-blindly divided into 4 groups of 22 people in each group (half male and half female), including (A) placebo group (2 sachets/day in 250 mL of water), (B) LY-66 probiotic group (7.5 × 10^9^ CFU/sachet, 2 sachets/day in 250 mL of water), (C) PL-02 probiotic group (7.5 × 10^9^ CFU/sachet, 2 sachets/day in 250 mL of water), (D) PL-02+LY-66 combined bacteria probiotic group 1:1 (7.5 × 10^9^ CFU/sachet, 2 sachets/day in 250 mL of water). All participants included in the study were first numbered according to the order of registration, and then, randomly assigned to four groups using a computer-generated random sampling method. The numbers were then redefined for identification purposes. Additionally, the sample manufacturer randomly assigned the codes A, B, C, and D to the four samples: placebo, PL-02, LY-66, and PL-02+LY-66. These coded samples were then distributed to the researchers who assigned them to the corresponding groups of participants. After the test and data analysis were completed, the sample manufacturer performed unblinding to reveal which samples corresponded to each group code. The probiotics were stored in a 4 °C environment and administered in a daily dosage of two sachets per intake, dissolved in 250 milliliters of water to prepare the beverage. This formulation, presented as a probiotic drink, served not only as a means of rehydration post-exercise but also facilitated the intake of probiotics. The intervention period lasted 6 weeks. The placebo contained anhydrous glucose, sodium citrate, lemon flavor, citric acid, sodium chloride, potassium citrate, sodium ascorbate, acesulfame potassium, and vitamin B complex. Probiotic-related products, anti-oxidant, anti-inflammatory drugs, or nutritional supplements were not allowed during the test. Subjects were required to photograph their meals on the day before the exercise test. Each photograph included a scale to facilitate accurate determination of portion sizes and nutritional content by the nutritionist. Additionally, subjects were asked to avoid extra exercise training from 48 h before and after the exercise test (Figure 1). The muscle-training regimen consisted of Tabata workouts conducted twice a week. Each session included the following exercises: (1) squats, (2) push-ups, (3) lunges, (4) high knees, (5) spiderman planks, (6) leg raises, (7) plank jacks, and (8) crunches. Each exercise was performed for 20 s followed by a 10 s rest. The complete Tabata workout comprised one set of these eight exercises. All subjects were required to train Tabata twice a week for a total of six weeks, a total of 12 times. The participation rate of each group was 100%, and no one was absent.

The relevant details of the experiment are as follows: the blood biochemistry, exercise tests, feces collection, maximum oxygen uptake, and body composition were tested before and after the intervention. Blood samples were collected 24 and 48 h after the exercise-induced muscle exhaustion for analysis of the inflammation damage indicator. Body composition was measured every two weeks. During the exercise test process, sport protectors were on hand to assist and guide subjects for avoiding sports injuries.

### 2.4. Body Composition Analysis

Body composition was assessed utilizing the InBody 570 device (InBody, Seoul, Republic of Korea), assessing total body weight, body fat percentage, muscle mass distribution, BMI, and basal metabolic rate. All assessments were conducted after participants had fasted for eight hours, following the methodology of a prior study [18].

### 2.5. Exercise Program to Induce Muscle Fatigue and Soreness

An exercise regimen involving 100 repetitive jumps was designed to induce muscle fatigue and soreness. Participants performed sets of 10 consecutive jumps, each to be completed within 4 s, followed by a 90 s rest period before the next set. This cycle was repeated until 100 jumps were completed. During each jump, the participant’s knees were required to be bent to a 90-degree angle to standardize the movement across all participants.

### 2.6. Countermovement Jump Assessment (CMJ)

Participants performed the CMJ on a Kistler force-measuring platform (9260AA, Kistler GmbH, Winterthur, Switzerland). They began by standing with hands on hips, performed a squat to a 90-degree knee bend, and then, jumped with maximum effort. Each participant completed three trials, with the platform calibrated to their individual weight to ensure accuracy. Measured parameters comprised the rate of force development (RFD), relative peak force, and jump height, offering detailed insights into each participant’s lower body power and force generation capabilities during the jumps.

### 2.7. Isometric Mid-Thigh Pull (IMTP)

The IMTP was conducted to assess maximal force generation using a custom-built IMTP rack (Kairos Strength, Murphy, NC, USA) and a force plate (Type 9260AA, Kistler, Winterthur, Switzerland), as per the protocol outlined in a prior study [21]. Participants were required to exert their maximum strength on the bar for 3–5 s, with tests repeated at 2 min intervals. The primary metrics recorded were peak force (the maximum strength exerted) and relative peak force (the maximum strength relative to body weight).

### 2.8. Wingate Anaerobic Test (WAnT)

The Wingate Anaerobic Test was conducted using the 894E anaerobic power bicycle (Monark Exercise AB, Dalarnas Lan, Sweden). Participants initially ramped up their cycling speed to 120 rpm, at which point a resistance equivalent to 7.5% of the participant’s body weight was automatically applied to the wheel. This test required participants to sprint at full strength for 30 s. Metrics assessed included relative mean power (W/kg), relative peak power (W/kg), and the fatigue index, calculated as the percentage drop from peak power to the lowest power level sustained [22].

### 2.9. Measuring VO_2max_

VO_2max_ was assessed using the Bruce maximal treadmill protocol, established in 1973 [23]. Participants wore a face mask connected to a gas analysis system and had electrocardiogram electrodes placed in appropriate locations. The test started at a velocity of 7.2 km/hr, progressively increasing by 1.8 km/hr every two minutes until exhaustion. Data collection commenced once the participant’s heart rate reached 170 beats per minute and continued until exhaustion, defined by meeting at least two of three criteria: maximal heart rate (220 minus age), a respiratory exchange ratio over 1.1, or a rating of perceived exertion (RPE) above 18.

### 2.10. Physiological Observation and Serum Biochemical Analysis

Venous blood samples were collected from participants following a minimum fasting period of 8 h, both before and after the intervention. Biochemical markers including AST, ALT, BUN, creatinine, uric acid, total protein, cholesterol profiles, and glucose levels were quantified utilizing a Hitachi 717 analyzer (Hitachi, Tokyo, Japan). Creatine kinase (CK) levels and IL-6 concentrations were determined using a Beckman Coulter AU5800 autoanalyzer (Beckman Coulter Inc., Brea, CA, USA) and an ELISA kit (R&D Systems, Inc., Minneapolis, MN, USA), respectively.

### 2.11. Fecal DNA Extraction and Next Generation Sequencing (NGS) Analysis

Participants provided a fecal sample before and after the experiment to examine alterations in the gut microbiota. DNA extraction was conducted from fecal samples following the experimental procedures outlined by the manufacturer of the QIAamp^®^ DNA Mini Kit (QIAGEN Canada, Mississauga, ON, Canada). After undergoing purification and extraction processes, the DNA was employed as a template for polymerase chain reaction (PCR). The specific primer pair employed in our PCR analysis comprised the forward primer 314F (5′-TCGTCGGCAGCGTCAGATGTGTATAAGAGACAGCCTACGGGNGGCWGCAG-3′) and the reverse primer 805R (5′-GTCTCGTGGGCTCGGAGATGTGTATAAGAGACAGGACTACHVGGGTATCTAATCC-3′). This primer set was devised for the selective amplification of the V3–V4 region within the bacterial 16S rRNA gene. The DNA amplification was conducted employing KAPA HiFi HotStart ReadyMix (Roche Sequencing Solutions, Pleasanton, CA, USA [KK2601]). Thermal cycling was performed using the following parameters: initially, the samples were denatured at 95 °C for 5 min. This was followed by 30 cycles, each consisting of denaturation at 95 °C for 30 s, annealing at 60 °C for 30 s, and extension at 72 °C for 30 s. Finally, an extension step was carried out at 72 °C for 5 min. To facilitate the construction of DNA libraries, the PCR products underwent processing with Nextera XT Index and Illumina sequencing adapters. The prepared DNA libraries underwent paired-end sequencing (2 × 300 bp) using an Illumina MiSeq platform (Illumina, San Diego, CA, USA), conducted by Majorbio Bio-Pharm Technology Co., Ltd. (Shanghai, China).

### 2.12. Bioinformatics Analysis and Statistics

We employed BaseSpace Sequence Hub (Illumina, San Diego, CA, USA) for the management of the sequenced data. The initial processing involved quality control and elimination of unpaired reads from the original paired-end sequences. These reads were subjected to clustering into operational taxonomic units (OTUs) using the May 2013 Greengenes taxonomic database for subsequent downstream analysis [24]. To investigate the diversity composition of intestinal microorganisms among distinct groups, alpha diversity and beta diversity were analyzed [25]. The diversity index was computed utilizing the ‘vegan’ package in the R statistical software environment; accessible at https://cran.r-project.org/, accessed on 20 December 2023. Alpha diversity pertains to the diversity within a particular geographical area or ecosystem, while beta diversity encompasses the diversity observed among different geographical areas or ecosystems. Shannon diversity has been employed as an assessment tool to gauge the richness of the community, wherein an elevated numerical value signifies increased abundance. The principal coordinate analysis (PCoA) figures depicting the beta diversity index were generated utilizing the ‘ggplot2’ R package. The Bray–Curtis matrix PERMANOVA testing was conducted employing the adonis function within the vegan package in R version 4.3.2. Pairwise comparisons were performed utilizing Student’s *t*-test, while analysis among multiple groups employed the same method. Permutational multivariate analysis of variance (PERMANOVA) was applied to assess statistical variances in beta diversity, utilizing QIIME2. A significance threshold of *p* < 0.05 was established for determining statistical significance.

### 2.13. Statistics

Statistical analyses were conducted using the SAS 9.0 software (SAS Institute, Cary, NC, USA). A one-way analysis of variance (ANOVA) was utilized to perform multi-group comparisons. Prior to conducting ANOVA, all data sets were assessed for normality. For data adhering to a normal distribution, the paired *t*-test was applied to evaluate differences between groups. In cases where data did not follow a normal distribution, the non-parametric Wilcoxon signed-rank test was employed. A *p*-value below 0.05 was considered statistically significant. All results are presented as mean ± standard deviation (SD).

## 3. Results

### 3.1. The Effects of Supplementing with PL-02, LY-66, or PL-02+LY-66 Probiotics Were Beneficial for Body Composition

Table 1 presents the body composition data of the subjects before and after six weeks of oral supplementation with PL-02, LY-66, and PL-02+LY-66 probiotics. In the placebo group, there were no significant differences observed in body weight (BW), body mass index (BMI), muscle mass, and fat body mass (FBM) values between the baseline and endpoint. Similarly, no significant differences were detected in either BW or BMI across all treatment groups before and after probiotic intervention. Regarding muscle mass, the initial mean muscle mass for the PL-02 group, LY-66 group, and PL-02+LY-66 group was 28.3 ± 7.1 kg, 27.7 ± 6.3 kg, and 30.0 ± 7.9 kg, respectively. After 6 weeks of intervention, the final mean muscle mass for the PL-02 group, LY-66 group, and PL-02+LY-66 group was 28.7 ± 7.0 kg, 27.9 ± 6.2 kg, and 30.0 ± 7.8 kg, respectively. Specifically, the PL-02 group showed a significant increase in muscle mass compared to baseline at 6 weeks of intervention (*p* < 0.05). Additionally, the initial mean body fat percentages of the PL-02 group, LY-66 group, and PL-02+LY-66 group were 21.2 ± 8.0%, 20.8 ± 8.1%, and 21.9 ± 6.5%, respectively. Following 6 weeks of intervention, the final mean body fat percentages for the PL-02 group, LY-66 group, and PL-02+LY-66 group were 20.4 ± 7.9%, 19.8 ± 8.0%, and 21.2 ± 6.2%, respectively. Notably, both the LY-66 group (*p* < 0.05) and PL-02+LY-66 group (*p* < 0.01) exhibited a significant reduction in body fat percentage compared to baseline at 6 weeks of intervention.

### 3.2. Effects of Biochemical Characteristics of Subjects before and after 6-Week Probiotic Intervention with PL-02, LY-66, or PL-02+LY-66

Prior to the intervention, all participants underwent baseline blood parameter analysis to assess their physiological state and to identify any potential adverse effects or side effects following the 6-week PL-02, LY-66, or PL-02+LY-66 intervention. The results, as presented in Table 2, indicate that liver function markers (AST, ALT), renal function markers (BUN, CREA, UA, TP), blood lipid profile (TC, TG, HDL-C, LDL-C), and blood glucose levels were all within the normal range before and after the intervention. No significant differences were observed among the groups pre- and post-intervention. Furthermore, there were no significant changes within each group after the intervention compared to before the intervention. Importantly, no adverse events associated with the intervention were reported throughout the study period.

### 3.3. Effect of PL-02, LY-66, or PL-02+LY-66 Probiotic Supplementation on Maximal Oxygen Consumption

**Figure 2:**
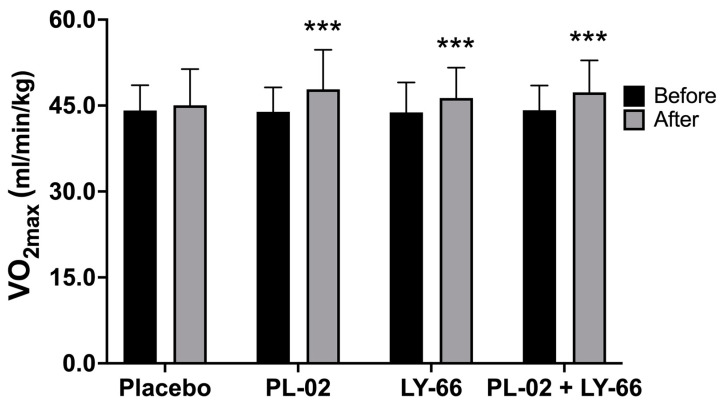
Comparing the effects of PL-02, LY-66, and PL-02+LY-66 on maximal oxygen uptake (VO_2max_) before and after 6 weeks of intervention. All values are expressed as mean ± SD. * indicates significant differences compared to the baseline within each group (*** *p* < 0.001).

Prior to the intervention, the baseline VO_2max_ values were recorded for the placebo group, PL-02 group, LY-66 group, and PL-02+LY-66 group as 44.1 ± 4.4, 43.9 ± 4.3, 43.8 ± 5.2, and 44.2 ± 4.3 mL/min/kg, respectively (Figure 2). After a 6-week intervention period, the placebo group exhibited a slight increase to 45.1 ± 6.3 mL/min/kg. In contrast, the PL-02 group, LY-66 group, and PL-02+LY-66 group demonstrated significant increases to 47.8 ± 6.9, 46.3 ± 5.3, and 47.3 ± 5.6 mL/min/kg for each respective group (Figure 2). Statistical analysis revealed significant improvements in VO_2max_ within the PL-02 group (increase of 1.09-fold, *p* < 0.001), LY-66 group (increase of 1.06-fold, *p* < 0.001), and PL-02+LY-66 group (increase of 1.07-fold, *p* < 0.001) post-intervention. Conversely, no significant change was observed in the placebo group’s VO_2max_ pre- and post-intervention.

### 3.4. Effect of PL-02, LY-66, or PL-02+LY-66 Probiotic Supplementation on Enhancing Strength Performance, Explosive Power, and Anaerobic Power

**Figure 3:**
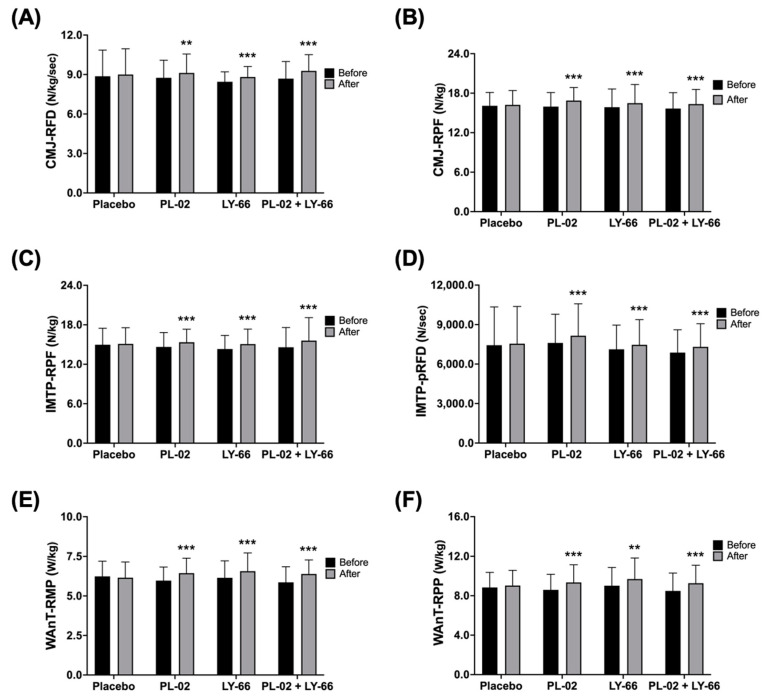
The effect of supplementation with PL-02, LY-66, or PL-02+LY-66 on athletic performance was evaluated using CMJ, IMTP, and WAnT tests. The CMJ test assessed performance in (**A**) RFD and (**B**) RPF before and after the intervention. The IMTP test assessed performance in (**C**) RPF and (**D**) pRFD before and after the intervention. Additionally, the WAnT test assessed performance in (**E**) RMP and (**F**) RPP before and after the intervention. All values are expressed as mean ± SD with a sample size of n = 22 for each group. * indicates a significant difference compared to before and after intervention within each group (** *p* < 0.01 and *** *p* < 0.001).

To investigate the effects of supplementing with PL-02, LY-66, or PL-02+LY-66 probiotics on strength performance, explosive power, and anaerobic power, participants from different treatment groups underwent 6 weeks of oral probiotic supplementation before testing. Firstly, we investigated the effects of probiotic supplementation on strength performance and explosive power in a countermovement jump (CMJ) test. The baseline rate of force development (RFD) values were recorded for the placebo group, PL-02 group, LY-66 group, and PL-02+LY-66 group as 8.9 ± 2.0, 8.8 ± 1.3, 8.5 ± 0.8, and 8.7 ± 1.3 N/kg/sec, respectively (Figure 3A). Following a 6-week intervention period, the placebo group exhibited a slight increase to 9.0 ± 2.0 N/kg/sec. In contrast, the PL-02 group, LY-66 group, and PL-02+LY-66 group demonstrated significant increases, with values rising to 9.1 ± 1.4, 8.8 ± 0.8, and 9.3 ± 1.2 N/kg/sec, respectively (Figure 3A). Notably, the RFD increased significantly, by 1.04-fold in the PL-02 group (*p* < 0.01), 1.04-fold in the LY-66 group (*p* < 0.001), and 1.07-fold in the PL-02+LY-66 group (*p* < 0.001) (Figure 3A). Baseline relative peak force (RPF) values were initially recorded for the placebo group, PL-02 group, LY-66 group, and PL-02+LY-66 group, yielding values of 16.1 ± 2.0, 16.0 ± 2.1, 15.9 ± 2.8, and 15.7 ± 2.4 N/kg, respectively (Figure 3B). Following a 6-week intervention period, the placebo group exhibited a marginal increase to 16.2 ± 2.2 N/kg. In contrast, the PL-02 group, LY-66 group, and PL-02+LY-66 group demonstrated significant enhancements to 16.9 ± 2.0, 16.5 ± 2.8, and 16.3 ± 2.2 N/kg, respectively (Figure 3B). Remarkably, the PL-02 group, LY-66 group, and PL-02+LY-66 group exhibited noteworthy increases in RPF, demonstrating respective increases of 1.06-fold (*p* < 0.001), 1.04-fold (*p* < 0.001), and 1.07-fold (*p* < 0.001) (Figure 3B).

Next, the impact on IMTP performance and explosiveness was assessed. The baseline RPF values were documented for the placebo group, PL-02 group, LY-66 group, and PL-02+LY-66 group as 15.0 ± 2.5, 14.6 ± 2.2, 14.3 ± 2.1, and 14.6 ± 3.0 N/kg, correspondingly (Figure 3C). Subsequent to a 6-week intervention period, the placebo group displayed a slight increase to 15.1 ± 2.5 N/kg. Conversely, the PL-02 group, LY-66 group, and PL-02+LY-66 group demonstrated notable increases, with values increasing to 15.4 ± 2.0, 15.1 ± 2.3, and 15.6 ± 3.5 N/kg, respectively (Figure 3C). Particularly, the PL-02 group, LY-66 group, and PL-02+LY-66 group exhibited respective increases of 1.05-fold (*p* < 0.001), 1.05-fold (*p* < 0.001), and 1.07-fold (*p* < 0.001) in RPF (Figure 3C). The baseline peak rate of force development (pRFD) values were documented for the placebo group, PL-02 group, LY-66 group, and PL-02+LY-66 group as follows: 7435.8 ± 2903.4, 7605.8 ± 2184.0, 7120.6 ± 1845.4, and 6870.8 ± 1735.2 N/s, correspondingly (Figure 3D). Subsequent to a 6-week intervention period, the placebo group displayed a modest increase to 7554.1 ± 2823.3 N/s. Conversely, the PL-02 group, LY-66 group, and PL-02+LY-66 group demonstrated notable increases, with values rising to 8156.9 ± 2421.6, 7466.4 ± 1903.9, and 7307.3 ± 1760.1 N/s, respectively (Figure 3D). Significant enhancements in pRFD were observed in the PL-02 group, LY-66 group, and PL-02+LY-66 group, with increases of 1.07-fold (*p* < 0.001), 1.05-fold (*p* < 0.001), and 1.06-fold (*p* < 0.001), respectively (Figure 3D).

Regarding anaerobic power performance, baseline body weight relative mean power (RMP) values were documented for the placebo group, PL-02 group, LY-66 group, and PL-02+LY-66 group at 6.2 ± 1.0, 6.0 ± 0.9, 6.2 ± 1.1, and 5.9 ± 1.0 W/kg, respectively (Figure 3E). Following a 6-week intervention period, the placebo group exhibited a sustained mean value of 6.2 ± 1.0 W/kg. In contrast, the PL-02 group, LY-66 group, and PL-02+LY-66 group exhibited notable increases, with mean values rising to 6.4 ± 1.0, 6.6 ± 1.2, and 6.4 ± 0.9 W/kg, respectively (Figure 3E). Specifically, the PL-02 group, LY-66 group, and PL-02+LY-66 group demonstrated respective increases of 1.08-fold (*p* < 0.001), 1.07-fold (*p* < 0.001), and 1.09-fold (*p* < 0.001) in RMP performance (Figure 3E). Baseline relative peak power (RPP) values were recorded for the placebo group, PL-02 group, LY-66 group, and PL-02+LY-66 group, yielding measurements of 8.8 ± 1.5, 8.6 ± 1.6, 9.0 ± 1.9, and 8.5 ± 1.8 W/kg, respectively (Figure 3F). Following a 6-week intervention period, the placebo group demonstrated a modest increase to 9.0 ± 1.6 W/kg. In contrast, the PL-02 group, LY-66 group, and PL-02+LY-66 group exhibited notable increases, with values increasing to 9.4 ± 1.8, 9.7 ± 2.1, and 9.3 ± 1.8 W/kg, respectively (Figure 3F). Correspondingly, RPP performance improved by 1.09-fold (*p* < 0.001), 1.08-fold (*p* < 0.01), and 1.09-fold (*p* < 0.001) for the PL-02 group, LY-66 group, and PL-02+LY-66 group, respectively (Figure 3F).

### 3.5. Effect of PL-02, LY-66, or PL-02+LY-66 Probiotic Supplementation on Restoring Muscle Performance and Explosive Strength after EIMD

**Figure 4:**
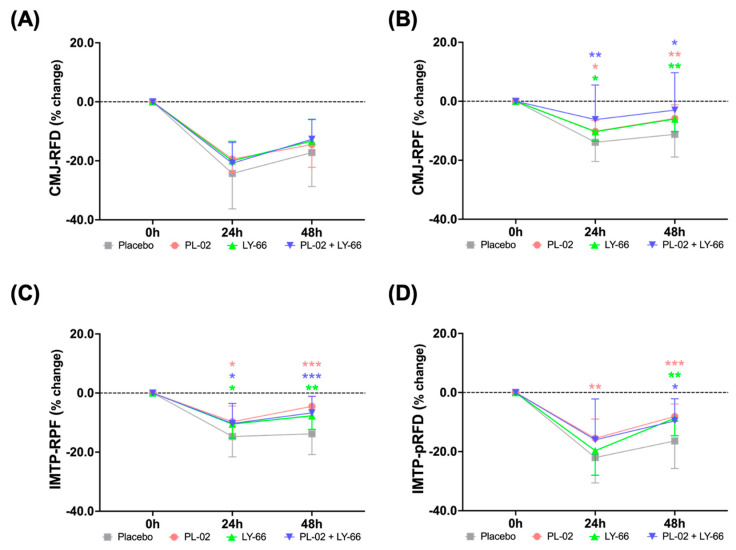
The efficacy of supplementation with PL-02, LY-66, or PL-02+LY-66 in mitigating strength loss was evaluated at 24 and 48 h post-EIMD using CMJ and IMTP tests. The CMJ test assessed performance in (**A**) RFD and (**B**) RPF at 24 and 48 h post-EIMD. The IMTP test assessed performance in (**C**) RPF and (**D**) pRFD at 24 and 48 h post-EIMD. All values are expressed as mean ± SD with a sample size of n = 22 for each group. ^*^ indicates a significant difference compared to placebo and treatment groups at same time points (* *p* < 0.05, ** *p* < 0.01, and *** *p* < 0.001).

To assess the benefits of PL-02, LY-66, or PL-02+LY-66 probiotic supplementation on CMJ and IMTP after EIMD, we investigated the extent of strength performance and explosive force reduction, as well as the recovery profiles at 24 and 48 h post-EIMD for different treatment groups following continuous 6-week probiotic intake prior to EIMD. We analyzed the percentage changes in RFD and RPF during CMJ observed at 24 and 48 h after the exhaustive exercise (Figure 4A,B). Upon comparing the post-EIMD measurements at 24 and 48 h between the placebo and probiotic groups, it was observed that probiotic groups exhibited a trend of acceleration in the recovery rate of RFD (Figure 4A). Regarding the RPF at 24 and 48 h post-EIMD, it was observed that the PL-02, LY-66, and PL-02+LY-66 groups demonstrated a significantly smaller decrease in RPF in comparison to the placebo group (*p* < 0.05) (Figure 4B).

We further investigated the percentage changes in IMTP and observed its RPF and pRFD at 24 and 48 h post-EIMD (Figure 4C,D). The PL-02, LY-66, and PL-02+LY-66 groups displayed a notable attenuation in the decline in RPF at 24 and 48 h post-EIMD compared to the control group (*p* < 0.05) (Figure 4C). At 48 h post-EIMD, these same treatment groups exhibited a significant increase in the recovery of RPF compared to the placebo group (*p* < 0.01). On the other hand, the PL-02, LY-66, and PL-02+LY-66 groups showed markedly lower levels of pRFD loss than the placebo group at 48 h post-EIMD (*p* < 0.05) (Figure 4D). Notably, the PL-02 group demonstrated significant protective effects in reducing the decline in pRFD compared to the placebo group at all post-EIMD time points (*p* < 0.01).

### 3.6. Effect of PL-02, LY-66, or PL-02+LY-66 Probiotic Supplementation on Blood Markers of Muscle Damage and Inflammation

**Figure 5:**
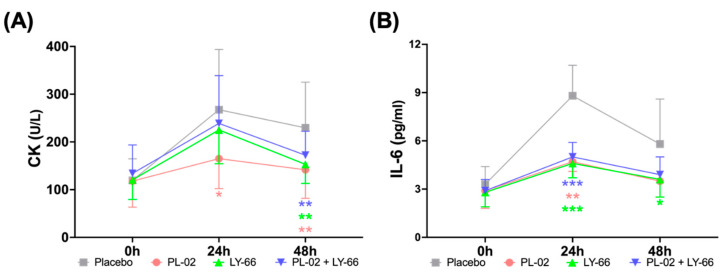
The effect of supplementation with PL-02, LY-66, or PL-02+LY-66 on blood markers were evaluated at 24 and 48 h post-EIMD, focusing on (**A**) creatine kinase (CK) levels, and (**B**) interleukin-6 (IL-6) concentrations. All values are expressed as mean ± SD with a sample size of n = 22 for each group. * indicates a significant difference compared to placebo and treatment groups at same time points (* *p* < 0.05, ** *p* < 0.01, and *** *p* < 0.001).

We proceeded to assess the effects of PL-02, LY-66, or PL-02+LY-66 probiotic supplementation on muscle damage and inflammatory markers following EIMD. Blood samples were collected from participants in each group, pre-EIMD and 24 and 48 h post-EIMD, after a 6-week probiotic supplementation period, for subsequent analysis. The activity percentage of the muscle damage marker CK increased in the placebo, PL-02, LY-66, and PL-02+LY-66 groups at 24 and 48 h after EIMD (Figure 5A). At 24 h post-EIMD, the PL-02 group exhibited a significant reduction in the CK activity compared to the placebo group (*p* < 0.05). Upon reaching the 48 h post-EIMD time point, the PL-02 group (*p* < 0.01), LY-66 group (*p* < 0.01), and PL-02+LY-66 group (*p* < 0.01) all demonstrated significant reductions in CK activity increase and accelerated recovery compared with the placebo group. The percentage changes in IL-6 concentration were significantly reduced at 24 h post-EIMD in the PL-02 group (*p* < 0.01), LY-66 group (*p* < 0.001), and PL-02+LY-66 group (*p* < 0.001) compared to the placebo group (Figure 5B). Additionally, at 48 h post-EIMD, the LY-66 group demonstrated a notable reduction in IL-6 concentration compared to the placebo group (*p* < 0.05). At all post-EIMD time points, the PL-02 group showed a notable decrease in CK concentration (*p* < 0.05), while the LY-66 group displayed significant efficacy in attenuating IL-6 levels (*p* < 0.05).

### 3.7. Effect of PL-02, LY-66, or PL-02+LY-66 Probiotic Supplementation on Modulation of Gut Microbiota

**Figure 6:**
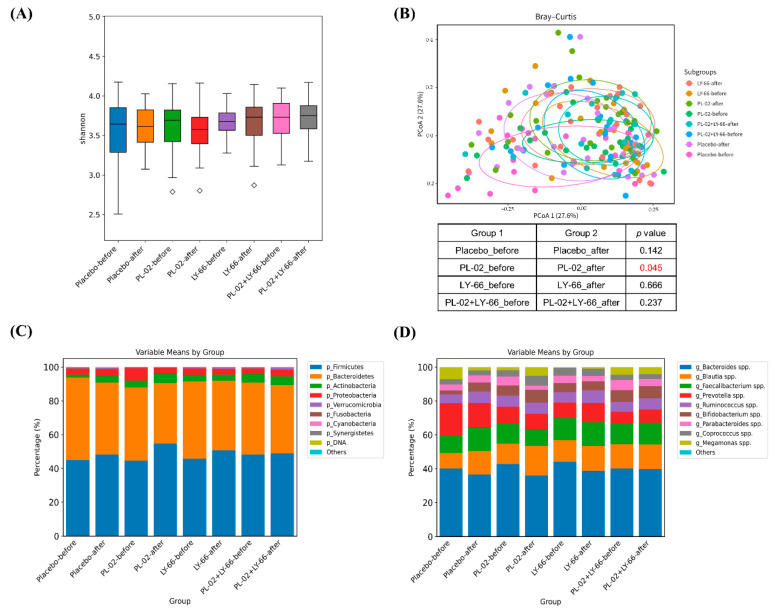
The alterations in the gut microbiota composition were evaluated both before and after a six-week period of supplementation with either PL-02, LY-66, or PL-02+LY-66 intervention. This assessment included (**A**) alpha diversity, (**B**) beta diversity, (**C**) the composition of the 10 most abundant phyla, and (**D**) genera. The red number in the (**B**) indicates a significant difference compared to before and after intervention within each group (*p* < 0.05).

To delve deeper into alterations in intestinal microbiota, we utilized next-generation sequencing (NGS) technology to analyze stool samples collected before and after a six-week period of probiotic intervention. Alpha diversity analysis revealed no significant differences across all groups (Figure 6A). Regarding beta diversity, while no significant alterations were observed in the placebo, LY-66, and PL-02+LY-66 groups, notable changes were detected in the PL-02 group (*p* < 0.05) (Figure 6B). Examination of gut microbiota composition at the phylum level revealed that Firmicutes, Bacteroidetes, Actinobacteria, and Proteobacteria comprised over 90% of the relative abundance in each group (Figure 6C). At the genus level, predominant taxa such as *Bacteroides*, *Blautia*, *Faecalibacterium*, *Prevotella*, and *Ruminococcus* collectively represented approximately 80% of the relative abundance across all groups (Figure 6D).

**Figure 7:**
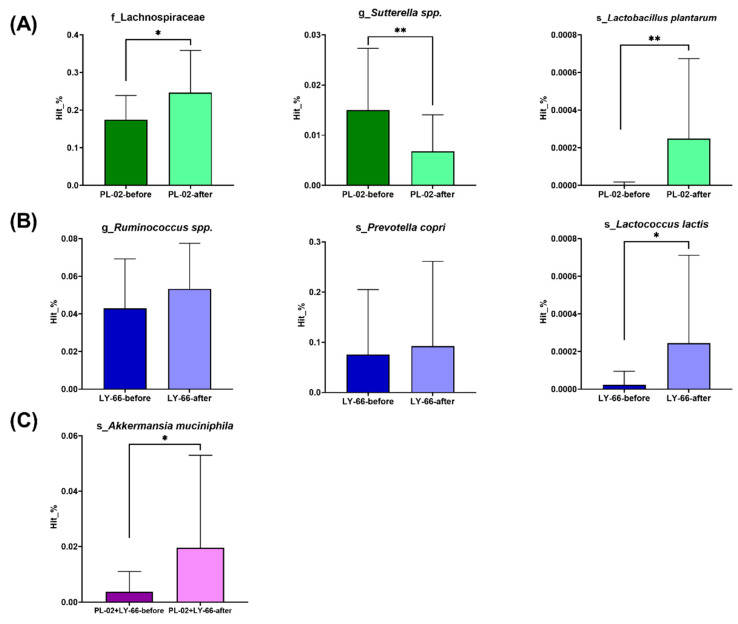
Following six weeks of supplementation with (**A**) PL-02, (**B**) LY-66, or (**C**) PL-02+LY-66, alterations in the microbiota were observed, specifically regarding changes in bacterial families, genera, and species. * The paired *t*-test was utilized to evaluate the differences in abundance within each group pre- and post-intervention: * *p* < 0.05 and ** *p* < 0.01.

Further analysis focused on specific bacterial populations within the intestinal microbiota. In the PL-02 group, there was a significant increase in the relative abundance of *Lactobacillus plantarum* (*p* < 0.01) and butyrate-producing *Lachnospiraceae* (*p* < 0.05) (Figure 7A). Additionally, a notable decrease in the pathobiont *Sutterella* was observed (*p* < 0.01) (Figure 7A). In the LY-66 group, trends toward an increase in *Ruminococcus* and *Prevotella copri* were noted, with a significant rise in the relative abundance of *Lactococcus lactis* (*p* < 0.05) (Figure 7B). The PL-02+LY-66 group showed a significant enhancement in the relative abundance of *Akkermansia muciniphila* (*p* < 0.05) (Figure 7C).

## 4. Discussion

Regular exercise is advantageous for enhancing human health by inducing increased muscle tension, enhancing posture, augmenting cardiovascular capacity, and elevating metabolic rates [26,27,28]. In recent years, the emphasis placed by health-conscious individuals on the potential applicability of probiotics in the development of sports nutrition supplements has witnessed a steady escalation. In this study, our objective was to investigate the potential benefits of probiotic supplementation in enhancing muscular performance. We enrolled a cohort of healthy non-athletic individuals and randomly assigned them into four groups, placebo, PL-02, LY-66, and PL-02+LY-66 supplementation, followed by a six-week intervention period. The results suggested that probiotic supplementation may contribute to improvement in exercise endurance and explosive strength, modulation of gut microbiota composition, reduction in circulating inflammatory biomarkers, mitigation of muscle damage, and acceleration of muscular recovery.

The public has consistently prioritized maintaining health, with recent research frequently linking changes in gut microbiota composition to various health conditions. As individuals age, a discernible reduction in the diversity of gut microbiota is often observed, typically coinciding with declines in skeletal muscle mass and function [29,30]. The importance of the gut microbiota in the growth and development of skeletal muscles has been validated in previous animal investigations. These investigations have delineated a correlation between gut microbiota and muscles, suggesting that a deficiency in gut microbiota may lead to loss in muscle mass [30,31]. For example, research conducted on rodents has demonstrated that germ-free (GF) mice, devoid of gut microbiota, exhibited muscle atrophy and reduced muscle mass compared to pathogen-free (PF) mice [32]. Similarly, GF piglets displayed a 40% decrease in lean body mass relative to conventional piglets [33]. Utilizing fecal microbiota transplantation, the introduction of gut microbiota into GF piglets not only successfully colonized the intestines but also partially restored muscle growth and development [33]. It has been hypothesized that the gut microbiota may influence the growth and function of host muscle tissue through two primary mechanisms: (1) by modulating the secretion of muscle-related hormones (such as insulin and IGF1) via activation of the gut–brain axis, and/or (2) by producing functional metabolic byproducts (such as SCFAs) that serve as signaling molecules in muscle cells [33,34,35,36,37].

This study noted an increase in beneficial bacteria such as *Lactobacillus*, *Akkermansia muciniphila*, and short-chain fatty acid (SCFA)-producing microbes following interventions with PL-02, LY-66, and PL-02+LY-66. Notably, in the PL-02 group, there was a significant increase in *Lactobacillus plantarum* and butyric acid-producing *Lachnospiraceae*, alongside a significant reduction in *Sutterella*. Butyrate, produced by microbial fermentation in the colon, offers numerous health benefits, including promoting bacterial equilibrium, attenuating inflammatory responses, and maintaining intestinal barrier integrity [38,39]. Moreover, SCFAs can activate AMP-activated protein kinase (AMPK) in muscle tissue by increasing the AMP/ATP ratio or through the Ffar2-leptin signaling pathway. This activation subsequently enhances protein synthesis in skeletal muscle [16,40]. In patients with inflammatory muscle diseases, a decrease in butyrate-producing bacteria has been observed [41], while butyrate synthesis by gut microbes positively influences skeletal muscle mass in healthy menopausal women [37].

The increased abundance of *Sutterella* within the intestinal microbiota has been identified as correlating with various human diseases, including inflammatory bowel disease (IBD) and asthma [42,43]. Asthma induces bronchial inflammation, triggering the accumulation of inflammatory cytokines (such as IL-4, IL-5, and IL-13) and free radicals [44,45]. This cascade ultimately results in the destruction of bronchial epithelial cells. Within the LY-66 group, *L. lactis* exhibited a significant increase, whereas *Ruminococcus* and *P. copri* demonstrated an upward trend. *Ruminococcus* is known for its role as a producer of SCFAs [46,47]. The increase in its relative abundance has been associated with a reduction in cardiovascular risk among obese individuals [47]. Elevating the abundance of *P. copri* in the human gastrointestinal tract is not only associated with a high-fiber diet but also with a reduction in visceral fat [48,49]. An animal study has shown that obese mice receiving prebiotic supplementation experienced an increase in *Prevotella* abundance alongside a decrease in fat mass [50]. Moreover, *A. muciniphila* was significantly increased in the PL-02+LY-66 group. Extensive research has consistently indicated an association between the depletion or decreased abundance of *A. muciniphila* and various diseases, including obesity, diabetes, hepatic steatosis, inflammation, and immune response to cancer therapy [51]. Briefly, our findings indicate that PL-02, LY-66, and PL-02+LY-66 each exhibit distinct efficacy in enhancing physical fitness. PL-02 demonstrated benefits in promoting muscle health, increasing muscle mass, and preventing conditions such as asthma. LY-66, on the other hand, primarily contributed to reducing body fat accumulation. PL-02+LY-66 effectively enhanced the colonization of *A. muciniphila* in the gut, indicating a comprehensive improvement in physical fitness. These probiotics all demonstrated efficacy in improving gut microbiota composition, with daily supplementation leading to a favorable shift in gut microbiota composition. Specifically, supplementation with the combination of PL-02 and LY-66 accelerated the improvement in gut microbiota composition.

The composition of the gut microbiota substantially influences systemic health. Observations from this study of improvements in body composition provide insight into the health benefits of probiotic supplementation. Following the probiotic intervention, significant enhancements were observed: the PL-02 group showed an increase in muscle mass (*p* < 0.05), while both the LY-66 and PL-02+LY-66 groups experienced notable reductions in body fat accumulation (*p* < 0.05). Furthermore, previous research has demonstrated that PL-02 supplementation significantly enhances muscle mass, strength, and endurance performance in mice (*p* = 0.0014) [52]. A systematic review by Prokopidis et al., covering 24 studies, indicated that probiotic supplementation could improve muscle mass relative to a placebo [53]. Additional studies have shown that probiotics, such as *L. sakei* CJLS03 and the MED-02 probiotic complex, effectively reduce body fat accumulation [54,55]. These findings suggest that long-term and moderate probiotic supplementation may be beneficial for increasing muscle mass and decreasing body fat. Importantly, unlike pharmaceutical interventions, probiotics are generally considered safe and devoid of adverse reactions.

VO_2max_ is measured through gas exchange analysis and sets the physiological upper limit for aerobic performance [4,56]. Currently, an effective training approach to enhance VO_2max_ involves the utilization of high-intensity interval training (HIIT), a method proven to rapidly improve cardiopulmonary fitness [57,58,59]. When the body can efficiently consume and utilize oxygen, physical activity becomes more achievable at a given intensity. Our findings indicate that supplementation with PL-02, LY-66, and PL-02+LY-66 leads to a significant increase of approximately 1-fold in VO_2max_ after 6 weeks of probiotic intervention compared to baseline (*p* < 0.001). Animal experimentation has confirmed that supplementation with *Saccharomyces boulardii* can increase their VO_2max_ and aerobic exercise capacity (*p* < 0.05), while a human trial has demonstrated a similar enhancement with the intake of the multi-strain probiotic SANPROBI BARRIER (Sanprobi Ltd. Szczecin, Poland) [60,61]. These investigations demonstrated that probiotics had positive effects on the participants’ body composition and cardiopulmonary health.

Various strategies have been proposed to enhance athletic performance, with probiotic supplementation emerging as a promising approach [62]. Athletic performance is influenced by a complex interplay of biological, psychological, and environmental factors [63]. Among these, muscular explosiveness is crucial for athletic success. In our study, we utilized three distinct assessment methods—CMJ, IMTP, and WAnT—to evaluate exercise performance before and after probiotic supplementation. Maximal rate of force development (RFD) and relative peak force are widely recognized as critical indicators for assessing muscle strength and power [11,64,65]. Our findings indicate that supplementation with PL-02, LY-66, and PL-02+LY-66 enhances muscular explosiveness, leading to significant improvements in athletic performance across all tests. These observations align with prior research findings, wherein male individuals participating in resistance training and soldiers enrolled in soldier self-defense courses demonstrated enhanced vertical jump performance following *B. coagulans* supplementation [66,67]. Similarly, athletes who received supplementation with yogurt containing *Streptococcus thermophilus*, *L. delbrueckii* subsp. *bulgaricus,* and *L. casei* CNCM-15182 showed improvements in vertical jump performance [68].

Additionally, we observed the effects of probiotic supplementation on mitigating EIMD and enhancing recovery. Research shows that muscle strength typically decreases within 24 to 48 h post-exercise and gradually returns to baseline after about 72 h [69,70]. Our results demonstrated that supplementation with PL-02, LY-66, and PL-02+LY-66 mitigated reductions in RPF and pRFD post-exercise, while also accelerating recovery of these parameters, thus supporting muscle protection and efficient recovery after physical exertion. Prior research has found that the administration of 1 billion CFU of *B. coagulans* GBI-30 6086 with 20 g of casein for two weeks significantly improved perceived recovery at 24 and 72 h after resistance exercise, as well as reducing muscle soreness at the 72 h mark [17]. In another investigation, the intake of Ultrabiotic 60™ and *S. boulardii* was observed to contribute to the alleviation of muscle soreness in elite male rugby players over a 17-week training and competition period [71].

The beneficial effects of probiotics on muscle health may stem from the influence of gut microbiota, potentially mediated through metabolic products such as SCFAs, secondary bile acids (BAs), and specific amino acids that modulate muscle function [29,62]. Protein or amino acid intake can activate the mammalian target of rapamycin (mTOR) pathway, enhancing muscle protein synthesis and supporting recovery after exercise [16]. Some studies have combined probiotics with pea protein, enhancing amino acid absorption [72,73]. Furthermore, research into the enhancement in muscle explosiveness through probiotics has also attracted attention. Over a 12-week period, the administration of *L. casei* Shirota (1 × 10^9^ CFU/mouse/day) to SAMP8 mice elicited alterations in gene expression associated with mitochondrial biogenesis in muscle, notably impacting PGC1α, SIRT1, NRF1, and TFAM [74]. This modulation served to mitigate mitochondrial alterations associated with aging. The upregulation of the AMPK/SIRT1/PGC1α signaling pathway, as evidenced by previous research, has been found to enhance mitochondrial functionality and increase ATP production [75]. PGC-1α4, an isoform of PGC-1α, formed a complex with nuclear receptor PPARβ after resistance exercise training (RET), resulting in subsequent effects on the expression of key glycolytic genes [76]. This cascade facilitated glycolysis and glucose uptake within muscle tissue. Hence, we postulate that supplementation with PL-02, LY-66, and PL-02+LY-66 may facilitate muscle recovery by improving the efficiency of protein absorption and utilization. However, further research is needed to validate this hypothesis.

Biological markers such as CK and IL-6 are essential for evaluating muscle damage and inflammation. CK levels typically rise after intense physical exertion, serving as an indirect indicator of skeletal muscle damage, while IL-6 is often used to assess inflammatory responses triggered by exercise [62,77,78,79]. Probiotic supplementation has shown promise in reducing the production of inflammatory biomarkers and mitigating unnecessary immune system activation [80]. Previous studies have demonstrated that probiotics, including PL-02, LY-66, and PL-02+LY-66, can mitigate the elevation of CK levels post-exercise and decrease IL-6 concentrations [52,81]. These findings suggest that probiotic supplementation offers protective effects against muscle damage and reduces the inflammatory response following EIMD. Moreover, research on PL-02 has shown reductions in lactate, BUN, blood ammonia, and CK levels following exercise in animal models. Additionally, studies involving *Lactobacillus plantarum* PS128 and a high-dose combination probiotic capsule have shown decreases in exercise-induced IL-6 concentrations [82,83]. Our study’s six-week supplementation period highlighted improvements in gut microbiota composition and overall gut health. In this study, we found that probiotic supplementation significantly improved markers related to muscle damage and inflammation, specifically CK and IL-6 levels. Although there may be deviations due to individual physiological differences among a small number of subjects, all participants in this study were students from the same university, of similar age groups, with the same daily routines and living habits. Additionally, the experimental intervention included the same exercise training for all subjects, and participants were required to refrain from exercise for two days before the exercise test. Therefore, despite some individual differences within the group leading to larger standard deviations, this does not affect the overall trend of improvement observed with probiotic supplementation. Looking ahead, further research should explore the potential benefits of prolonged probiotic supplementation and conduct in-depth studies on specific changes in gut microbial ratios. These investigations are crucial for understanding the cellular and molecular mechanisms underlying probiotics’ influence on muscle health and fatigue recovery.

This study has several limitations: First, all participants were non-athletic majors from the same university. Although their living conditions were similar, reducing variability, the results may not be generalizable to a broader population. Secondly, the primary focus of this study was to compare the effects of different probiotics on muscle mass and exercise performance, necessitating the inclusion of both male and female subjects in the same group for comparison. Therefore, future trials should include a larger sample size to more thoroughly compare the effects and mechanisms in both sexes. Third, our study subjects were non-professional athletes whose physical activity levels were relatively consistent. Given that all subjects received Tabata training during the study period, we considered it feasible to disregard their individual athletic background. Furthermore, due to the lack of professional nutritionists in our research team, we were unable to conduct a comprehensive analysis of the dietary intake of our subjects. However, we recognize the importance of collecting comprehensive data on participants’ physical fitness levels, diet, and other lifestyle factors, as these variables can significantly influence our study results. In future research, we aim to incorporate these factors to better understand their impact and increase the robustness of our findings. Fourth, currently, there are no studies that clearly delineate the effectiveness of the probiotic supplementation period in improving sports performance. Despite this, we referenced the past literature and provided probiotic supplementation for over six weeks. However, further research is needed to compare whether longer-term interventions yield greater benefits. Fifth, additionally, given the extensive testing in this experiment, we limited data collection to 24 and 48 h after EIMD to consider the burden on participants. However, more significant changes might be observed over extended periods, including both the elevation and recovery phases. For instance, CK levels peak at 24–48 h, and numerous studies have documented recovery conditions beyond 72 and 96 h [84]. Therefore, more in-depth investigations into key parameters over longer durations are necessary for future research.

## 5. Conclusions

Our study demonstrates that six weeks of a continuous combination of exercise training and supplementation with PL-02, LY-66, and PL-02+LY-66 significantly enhances muscle strength, explosive power, and endurance performance. Probiotic supplementation was also found to effectively attenuate the decline in muscle strength and explosive power following EIMD, reduce the elevation of inflammatory markers in the blood, and accelerate the recovery process. Notably, the intake of PL-02 significantly increased muscle mass, while the consumption of LY-66 and the combined PL-02+LY-66 formulation led to a substantial reduction in body fat percentage. Additionally, supplementation with these probiotics resulted in the modulation of gut microbiota, increasing the prevalence of beneficial bacteria. These findings highlight that the combination of exercise training and supplementation with probiotics not only enhances exercise performance but also improves the management of exercise-induced muscle damage and recovery. Future research should delve deeper into the mechanisms by which PL-02, LY-66, and PL-02+LY-66 aid in muscle damage recovery, to further elucidate the role of probiotics in enhancing athletic performance. This will expand our understanding of how probiotics can be effectively integrated into sports nutrition strategies.

## Figures and Tables

**Table 1:** Body composition profiles.

	Placebo		PL-02		LY-66		PL-02+LY-66	
Age (years)	21.9 ± 1.4		21.3 ± 1.5		21.1 ± 2.1		20.9 ± 2.0	
Body composition	Baseline	Endpoint	Baseline	Endpoint	Baseline	Endpoint	Baseline	Endpoint
Body weight (kg)	68.2 ± 12.7	68.1 ± 13.1	64.1 ± 13.6	64.0 ± 13.9	62.6 ± 12.4	62.5 ± 15.7	68.1 ± 16.0	67.7 ± 15.7
BMI (kg/m^2^)	23.7 ± 2.8	23.6 ± 3.1	22.5 ± 3.1	22.5 ± 3.2	22.0 ± 3.2	21.9 ± 3.3	23.2 ± 3.3	23.0 ± 3.3
Muscle mass (kg)	30.1 ± 6.5	29.9 ± 6.0	28.3 ± 7.1	28.7 ± 7.0 *	27.7 ± 6.3	27.9 ± 6.2	30.0 ± 7.9	30.0 ± 7.8
FBM (%)	21.8 ± 6.6	21.7 ± 7.0	21.2 ± 8.0	20.4 ± 7.9	20.8 ± 8.1	19.8 ± 8.0 *	21.9 ± 6.5	21.2 ± 6.2 **

All values are expressed as mean ± SD. * indicates significant differences within each group compared to their baseline (* *p* < 0.05 and ** *p* < 0.01). BMI, body mass index; FBM, fat body mass.

**Table 2:** Routine blood biochemical parameters of subjects before (Week 0) and after (Week 6) probiotic intervention.

Parameters	Week	Placebo	PL-02	LY-66	PL-02+LY-66
AST (U/L)	Week 0	22 ± 4	23 ± 4	22 ± 4	21 ± 4
	Week 6	21 ± 4	20 ± 5	19 ± 3	19 ± 3
ALT (U/L)	Week 0	20 ± 3	20 ± 4	21 ± 5	22 ± 4
	Week 6	18 ± 3	18 ± 4	18 ± 5	19 ± 4
TC (mg/dL)	Week 0	179 ± 26	173 ± 20	179 ± 25	174 ± 22
	Week 6	165 ± 21	169 ± 26	168 ± 23	170 ± 25
TG (mg/dL)	Week 0	67 ± 19	69 ± 19	68 ± 20	66 ± 18
	Week 6	63 ± 17	62 ± 18	65 ± 17	62 ± 14
HDL (mg/dL)	Week 0	66.6 ± 11.7	66.6 ± 12.6	67.3 ± 14.8	63.8 ± 10.7
	Week 6	60.7 ± 10.7	63.0 ± 9.3	65.0 ± 13.8	65.0 ± 13.8
LDL (mg/dL)	Week 0	94.5 ± 16.4	93.4 ± 19.6	91.4 ± 18.6	90.0 ± 12.0
	Week 6	88.3 ± 16.1	86.2 ± 17.6	85.4 ± 17.9	86.0 ± 10.6
BUN (mg/dL)	Week 0	14.9 ± 2.9	14.7 ± 3.2	14.7 ± 4.0	14.7 ± 3.3
	Week 6	14.3 ± 3.4	14.0 ± 2.7	14.3 ± 3.7	14.2 ± 3.3
CREA (mg/dL)	Week 0	0.98 ± 0.24	0.94 ± 0.22	0.97 ± 0.17	0.98 ± 0.19
	Week 6	0.96 ± 0.19	0.99 ± 0.16	0.98 ± 0.10	0.98 ± 0.12
TP (g/dL)	Week 0	7.24 ± 1.15	7.05 ± 1.01	7.10 ± 1.21	7.04 ± 1.17
	Week 6	7.62 ± 1.05	7.66 ± 0.84	7.65 ± 0.76	7.45 ± 0.71
UA (mg/dL)	Week 0	4.60 ± 0.96	4.25 ± 1.31	4.46 ± 1.41	4.70 ± 1.33
	Week 6	4.60 ± 0.76	4.54 ± 1.00	4.80 ± 1.31	4.77 ± 1.08
Glucose (mg/dL)	Week 0	82 ± 12	81 ± 9	86 ± 15	82 ± 11
	Week 6	84 ± 9	84 ± 7	84 ± 13	82 ± 6

All values are expressed as mean ± SD. AST, aspartate aminotransferase; ALT, alanine aminotransferase; TC, total cholesterol; TG, triacylglycerol; HDL, high-density lipoprotein; LDL, low-density lipoprotein; BUN, blood urea nitrogen; CREA, creatinine; TP, total protein; UA, uric acid.

## Data Availability

The data presented in this study are available within the article.
